# Explorations of Alaska Native Urban Eldership

**DOI:** 10.1093/geroni/igaa057.3366

**Published:** 2020-12-16

**Authors:** Steffi Kim

**Affiliations:** University of Alaska Anchorage, Anchorage, Alaska, United States

## Abstract

Successful aging in Alaska Native people or “Eldership” is a state that is embedded within a cultural, relational, and generational context (Boyd, 2018; Lewis, 2011; Wexler, 2014). Eldership has been described as a developing and nuanced personal quality shaped by individual, relational, and contextual influences (Kim, 2020). Within the cultural and traditional understanding of Alaska Native people, the concept of Eldership is analogous to the Western concept of successful aging. With increasing numbers of outmigration from rural community members (Driscoll et al., 2010), this study investigated the impact of relocation from a rural traditional community to an urban Western community and its effect on the Elder’s perception of “Eldership.” This study considered the broader impact of the multi-systemic and socio-ecological context of minority and majority culture, dominant culture, and its implications for successful aging and identity (Grandbois & Sanders, 2009; Kirmayer et al., 2011). Gee’s discourse analysis was used for the systematic interpretative study of 25 Elder interviews regarding their use of language describing their lived experiences, including three identified discursive patterns: cultural discourse, psychological discourse, and Elder identity discourse. Based on the study findings, Elders’ experiences are impacted by socio-economical and cultural differences encountered during relocation shifting the cultural frame of Alaska Native “Eldership” according to rural or urban contexts.

